# Botanical Origins and Antioxidant Activities of Two Types of Flavonoid-Rich Poplar-Type Propolis

**DOI:** 10.3390/foods12122304

**Published:** 2023-06-07

**Authors:** Jiangtao Qiao, Yingying Wang, Yu Zhang, Lingjie Kong, Hongcheng Zhang

**Affiliations:** 1 State Key Laboratory of Resource Insects, Institute of Apicultural Research, Chinese Academy of Agricultural Sciences, Beijing 100093, Chinazhanghongcheng@caas.cn (H.Z.); 2 Jiangsu Beevip Biotechnology Co., Ltd., Taizhou 225300, China; 3 Key Laboratory of Bee Products for Quality and Safety Control, Ministry of Agriculture and Rural Affairs, Beijing 100093, China

**Keywords:** poplar-type propolis, phenolic composition, botanical origin, antioxidant activity, flavonoids

## Abstract

(1) Background: Propolis has attracted attention in recent years due to its important pharmacological effects. The present study aimed to investigate the botanical origins of 39 propolis samples and evaluate their antioxidant activities; (2) Methods: A HPLC-PDA system was used to analyze the phenolic compositions of propolis and poplar bud resin samples. The antioxidant activities of propolis samples were evaluated by oxygen radical absorption capacity (ORAC) and superoxide anion free radical scavenging capacity assay; (3) Results: Our study shows that 17 propolis samples were characterized by five predominant flavonoids, including 5-methoxy pinobanksin, pinobanksin, pinocembrin, pinobanksin-3-acetate, and chrysin, while 22 propolis samples were characterized by four flavonoids (pinobanksin, pinocembrin, pinobanksin-3-acetate, and chrysin). The average contents of characteristic flavonoids reached up to over 70% and 65% of total phenolics, respectively. Furthermore, the botanical origins of the two types of propolis samples were identified as *Populus* × *euramericana* cv. ‘Neva’ and *Populus Simonii* × *P*. *nigra*, respectively; (4) Conclusions: Most notably, our results reveal that these propolis samples presented excellent antioxidant activities due to their high contents of flavonoid. These flavonoid-rich propolis samples can thus be used to develop low-allergen and high-antioxidant nutraceuticals.

## 1. Introduction

In the last few decades, researchers worldwide have shown an increasing interest in propolis. Propolis is a resinous substance that honeybees (*Apis mellifera* L.) collect from tree buds, leaves, and tissue-wound exudates around the hive, and then mix with beeswax, pollen, and volatiles [1,2]. Propolis consistently plays a crucial role in sealing hive walls, reinforcing the borders of combs, embalming dead intruders, and protecting honeycomb against pathogenic microorganisms. Nowadays, propolis is widely used as a natural preservative, an alternative medicine, a food supplement, and a cosmetic product in many countries. The popularity of propolis is due to its diverse chemical constituents, including flavonoids, phenolic acid and their esters, ketones, and terpenes. The plentiful constituents lessen the complexity of botanical sources collected by honeybees in a habitat [3]. Therefore, it is significant to assure the botanical origin of propolis.

Around the world, propolis can be divided into eight categories based on their botanical sources and chemical constituents: Poplar-type propolis from temperate regions; Aspen propolis from northern regions of Europe; Brazilian green propolis from southeastern Brazilian; South American red propolis from Cuba and Brazil; Mediterranean propolis from the Mediterranean region; Pacific propolis from Pacific islands; Mangifera indica propolis from Indonesian; and Mixed propolis, such as aspen-poplar and Pacific-Mangifera indicia propolis [4]. Poplar-type propolis is one of the most widespread types and is primarily found in Europe, North America, and non-tropical regions of Asia. In recent decades, China has emerged as the largest propolis producer in the world, and many researchers have speculated that the major plant source of Chinese propolis may be Populus species [5,6], based on the phytochemicals of bud resin from various Populus species. Populus can be divided into five different sections: *Tacamahaca* Spach, *Leuce* Duby, *Aigeiros* Duby, *Turanga* Bunge, and *Leucoides* Spach [7]. In Europe and North America, *Populus nigra* L. from the *Aigeiros* Duby section has been verified as the major botanical source of propolis [8,9]. However, in China, the widespread distribution of poplar trees and their several varieties seem to have led to the diverse botanical origins of Chinese propolis. On the other hand, a growing body of research has shown that the disease-preventing capacity of propolis can be attributed to its biological characteristics, especially with respect to powerful antioxidant activity. However, the chemical composition of propolis varies among different geographic areas, resulting in varying levels of biological properties, such as antioxidant activity. Extensive research has been conducted on the botanical origins and antioxidant activities of propolis collected from various countries and regions. Nonetheless, only a few studies on Chinese propolis have been published.

In this study, we collected 39 propolis and 5 poplar bud resin samples from different provinces across China. We utilized high-performance liquid chromatography with a photo-diode array (HPLC-PDA) to analyze the phenolic composition of the samples. To determine the botanical origin, we compared the chromatographic similarities between the propolis and poplar bud resin samples using the Similarity Evaluation System for Chromatographic Fingerprint of TCM. Additionally, we tested the antioxidant activities of the propolis samples using the oxygen radical absorbance capacity (ORAC) and superoxide anion free radical scavenging capacity assay.

## 2. Materials and Methods

### 2.1. Reagents and Chemicals

The following were purchased from Sigma-Aldrich Co. (St. Louis, MO, USA): 3,4-Dihydroxybenzylaldehyde (1), caffeic acid (2), vanillin (3), *p*-coumaric acid (4), ferulic acid (5), isoferulic acid (6), benzoic acid (7), 3, 4-dimethoxy cinnamic acid (8), cinnamic acid (9), 4-methoxy cinnamic acid (10), quercetin (13), alpinetin (14), kaempferol (15), apigenin (17), isorhamnetin (18), pinocembrin (19), benzyl caffeate (20), chrysin (22), phenethyl caffeate (23), galangin (24), benzyl *p*-coumarate (25), benzyl ferulate (26), pinostrobin (28), tectochrysin (29), cinnamyl cinnamate (31), trolox (6-hydroxy-2,5,7,8-tetramethylchroman-2-carboxylic acid), randomly methylated β-cyclodextrin (RMCD), fluorescein and 2,2′-Azobis-(2-amidinopropane) dihydrochloride (AAPH), luminol, pyrogallol, sodium bicarbonate, and sodium carbonate. Cinnamylideactic acid (16) was purchased from Funakoshi Chemical Co. (Tokyo, Japan), while 5-Methoxy pinobanksin (11), pinobanksin (12), pinobanksin-3-acetate (21), cinnamyl caffeate (27), cinnamyl *p*-cinnamate (30), and 4-methoxycinnamyl cinnamate (32) were collected by preparative HPLC.

Ethanol, acetone, dimethyl sulphoxide (DMSO), sodium hydroxide, potassium dihydrogen phosphate, and dipotassium phosphate (Analytical reagent grade) were purchased from Beijing Chemical Works (Beijing, China). Methanol and acetic acid in HPLC grade were obtained from Fisher Scientific (Pittsburgh, PA, USA). Ultrapure water was purified by using a Milli-Q-Integral System (Merk Millipore, MA, USA).

### 2.2. Propolis and Poplar Bud Resin Samples

We established two apiaries: one in Xinxiang city and another in Yichun city through the Modern Agro-industry Research System. In August 2019, we collected two propolis samples (S1 and S18) from the hives located at the two apiaries. Additionally, we collected five poplar bud samples within a six-kilometer radius of these apiaries during the same time period. The poplar taxonomy was identified by the plant taxonomist Prof. Zhibin Luo from the Chinese Academy of Forestry. The poplar bud sample at the Xinxiang city apiary was identified as *Populus* × *euramericana* cv. ‘Neva’ (B1). Additionally, the remaining four poplar bud samples at the Yichun city apiary were identified as *Populus ussuriensis* Kom. (B2), *Populus koreana* Rehd. (B3), *Populus cathayana* Rehd. (B4), and *Populus Simonii* × *P*. *nigra* (B5), respectively. The collected poplar bud samples were frozen at −80 °C until resin on the bud surface became stiff solid. After that, the bud resins were peeled away from the surface of the poplar buds carefully by tweezers.

In addition, we also obtained 37 other propolis samples (S2 to S17, S19 to S39) from beekeepers in 18 different provinces across China. These samples were collected from June to August 2019, and the location information of each sample can be found in the Supplementary Materials (Figure S1). All 39 propolis and 5 poplar bud resin samples were stored in a refrigerator at −18 °C until analysis.

### 2.3. Preparation of Propolis and Poplar Bud Resin Samples for HPLC Analysis

The frozen propolis or poplar bud resin samples (0.5 g) were cut into pieces and pulverized using a mill (XFB-500, Zhongzhou Co., Chongqing, China), followed by the extraction of all samples using 10 mL of solvent (75% ethanol/water, *v*/*v*) for 3 h under ultrasonication (DCTZ-1000, Hongxianglong Biotechnology Co., Ltd., Beijing, China) at a frequency of 40 kHz and power of 100 W. The resulting mixtures were allowed to stand at room temperature for 12 h. Finally, the supernatant was filtered using MILLEX-GA 0.22 μm filter for HPLC-PDA analysis.

### 2.4. Preparation of Propolis Samples for Antioxidant Activity Analysis

In accordance with Section 2.3, the resulting powdered propolis sample (10 g) was then extracted using an ultrasonic extract with 200 mL of solvent for 4 h. The resulting suspensions were then centrifuged at 12,000× *g* for 30 min to obtain supernatants, which were concentrated using a rotary evaporator (Rotavapor, R-215, Buchi Co., Ltd., Flawil, Switzerland) and finally freeze-dried.

### 2.5. Phenolics Analysis of Propolis and Poplar Bud Resin Samples by HPLC-PDA

To investigate the phenolic characteristics between propolis and poplar bud resin samples, all supernatants of extracts were determined by LC-6AD high-performance liquid chromatography (Shimadzu, Tokyo, Japan), which included a photodiode array detector (PDA), a vacuum degasser, a quaternary pump, a SIL-Automatic sampler, a CTO-10A column oven set at a steady temperature of 35 °C, and LC-solution chromatography software (version 5.96) for equipment control. The reversed-phase column Gemini C18 (150 × 4.6 mm, 3 μm) (Phenomenex, Inc., Torrance, CA, USA) was used for phenolics separation. The mobile phase consisted of water (Phase A) and methanol (Phase B), containing 2% acetic acid. A 150 min linear gradient with a flow rate of 0.65 mL/min was programmed as follows: 0–10 min, 22–32% B; 10–25 min, 32–35% B; 25–35 min, 35–38% B; 35–52 min, 38–51% B; 52–70 min, 51–52% B; 70–80 min, 52–52% B; 80–90 min, 52–53% B; 90–100 min, 53–59% B; 100–115 min, 59–63% B; 115–130 min, 63–75% B; 130–150 min, 75–80% B. The 10 μL supernatants were injected. The phenolic compounds were identified by comparing their retention time and UV spectra with commercial standards [10]. External calibration curves were used to quantify a total of 32 phenolic compounds at a wavelength of 280 nm.

### 2.6. Antioxidant Activity Analysis

#### 2.6.1. Hydrophilic and Lipophilic ORAC Assay

Hydrophilic and lipophilic ORAC assays were conducted with some modifications described in the literature [11]. In accordance with Section 2.4, propolis samples were extracted using five different solvents, including 75% ethanol (75% ethanol/water, *v*/*v*), water, ethyl acetate, diethyl ether, and petroleum ether. The dried extracts obtained from 75% ethanol or water were redissolved using DMSO and then further diluted with phosphate buffer (pH 7.4) to prepare the experimental concentrations for the hydrophilic ORAC assay. The dried extracts obtained from ethyl acetate, diethyl ether, or petroleum ether were first redissolved in 50% acetone/water (*v*/*v*) and then further diluted with 7% RMCD solution (50% acetone/50% water, *v*/*v*) for the lipophilic ORAC assay.

To perform the hydrophilic ORAC assay, we added 20 μL of the diluted sample extract solution, blank (phosphate buffer, pH 7.4), or Trolox calibration solution (phosphate buffer, 6.25, 12.5, 25, 50, 100 μmol/L) to a well in a 96-well bottom reading microplate. Then, we added 150 μL of fluorescein solution (8.16 × 10^−2^ μmol/L) to each well and incubated the plate at 37 °C for 10 min. Finally, we added 30 μL of AAPH solution (153 mmol/L) to each well as a peroxyl generator to start the reaction. We measured the fluorescence of each well using a microplate reader (Synergy HT, BioTek Instruments, Inc., Winooski, VT, USA) at 1 min intervals for a total of 50 min. We used software Gen 5^TM^ to measure fluorescence with an excitation wavelength of 485 nm and an emission wavelength of 528 nm. For the lipophilic ORAC assay, we used 7% RMCD solution as a blank and to prepare the Trolox calibration solution (6.25, 12.5, 25, 50, 100 μmol/L). We followed the same procedure for adding fluorescein solution and AAPH solution and measuring fluorescence as in the hydrophilic ORAC assay described above.

The final hydrophilic and lipophilic ORAC values were calculated by using a linear regression model (y = ax + b) between Trolox concentration (μmol) and the net area under curve (AUC) of the fluorescein decay curve. Linear regression was used in the range of 6.25–100 μmol Trolox. The ORAC values were expressed as micromoles of Trolox equivalents (TE) per 100 g of propolis sample (μmol TE/100 g). The AUC was calculated using the following equation based on Zhang [12]:

AUC = 0.5 + f_1_/f_0_ + f_2_/f_0_ + … + f_49_/f_0_ + 0.5(f_50_/f_0_)

where f_0_ is the initial fluorescence reading at 0 min, and f_50_ is the final fluorescence reading at the 50th minute. The net AUC was obtained by subtracting the AUC of the blank from that of a sample.

#### 2.6.2. Superoxide Anion Free Radical Scavenging Capacity Assay

The abilities of propolis samples to scavenge the superoxide anion free radical were evaluated using a chemiluminescence technique in the pyrogallol-luminol system [13]. A 6.25 × 10^−4^ mol/L pyrogallol solution was prepared with 0.1 mmol/L hydrochloric acid as a solvent, and a 1.0 mmol/L luminol solution was prepared with carbonate buffer solution (pH 10.2) for later use. Propolis samples were extracted using 75% ethanol as a solvent in accordance with Section 2.4. The dried extracts were then redissolved in DMSO and further diluted with carbonate buffer solution (pH 10.2) to create various concentrations (0.125, 0.25, 0.5, 1, 2, 4 mg/mL). A 30 μL sample of the diluted extract was mixed with 10 μL of pyrogallol and 960 μL of luminol solution to yield a final volume of 1000 μL through shaking. The emission light intensity was monitored at 25 °C every 6 s using a BPCL ultra-weak luminescence analyzer (Institute of Biophysics, Chinese Academy of Science, Beijing, China), and the total integral of the light intensity was recorded for 240 s. A control was conducted using 75% ethanol instead of the sample solution in the same manner, with Trolox serving as a positive control. Superoxide anion radical scavenging capacity was calculated using the following equation, and half inhibition concentrations (IC_50_) value was conducted to determine the antioxidant activity:

I(%) = 100 × (I_control_ − I_sample_)/I_control_

In this equation, I_sample_ is the emission intensity of the sample solution, and I_control_ is the emission intensity of the reaction with 75% ethanol.

### 2.7. Statistical Analysis

For statistical analysis, all experiments were performed in triplicate. The values were presented as means ± standard deviation and analyzed with SPSS software (version 16.0, SPSS GmbH Software, Munich, Germany). The IC_50_ values (the concentration of a sample that is required for 50% inhibition in vitro) were determined using linear regression. The chromatographic similarities of propolis and poplar bud resins were investigated using the Similarity Evaluation System for Chromatographic Fingerprint of TCM (Chinese Pharmacopoeia Committee, 2004A). Nine common peaks were selected to match similarities between the propolis samples and poplar bud resin samples.

## 3. Results

### 3.1. Characteristics of 17 Propolis Samples and Bud Resin of Populus × euramericana cv. ‘Neva’

**Figure 1:**
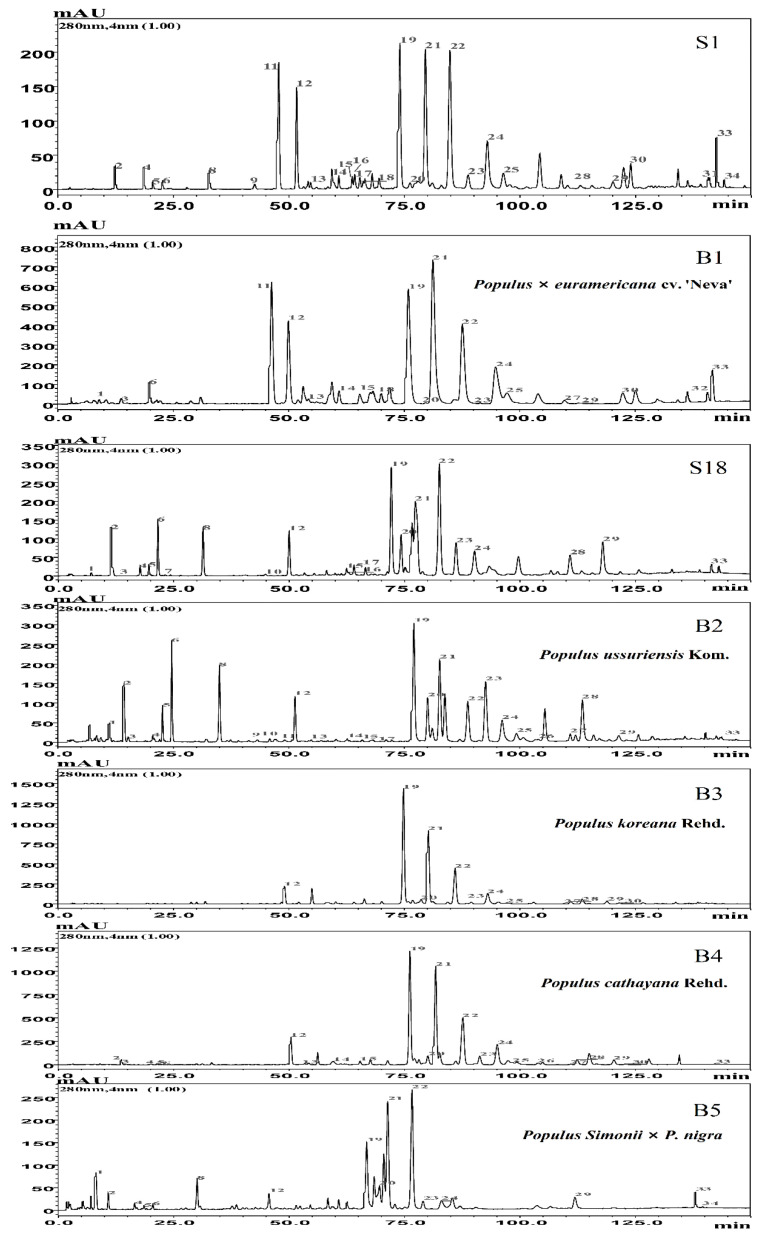
HPLC chromatograms of propolis sample S1 (Henan, Xinxiang), S18 (Heilongjiang, Yichun), and five bud resins. **Note:** (1) 3,4-Dihydroxybenzyl aldehyde; (2) Caffeic acid; (3) Vanillin; (4) *p*-Coumaric acid; (5) Ferulic acid; (6) Isoferulic acid; (7) Benzoic acid; (8) 3, 4-Dimethoxy cinnamic acid; (9) Cinnamic acid; (10) 4-Methoxy cinnamic acid; (11) 5-Methoxy pinobanksin; (12) Pinobanksin; (13) Quercetin; (14) Alpinetin; (15) Kaempferol; (16) Cinnamylideactic acid; (17) Apigenin; (18) Isorhamnetin; (19) Pinocembrin; (20) Benzyl Caffeate; (21) Pinobanksin-3-acetate; (22) Chrysin; (23) Phenethyl caffeate; (24) Galangin; (25) Benzyl *p*-coumarate; (26) Benzyl ferulate; (27) Cinnamyl caffeate; (28) Pinostrobin; (29) Tectochrysin; (30) Cinnamyl p-cinnamate; (31) Cinnamyl cinnamate; (32) 4-Methoxy cinnamyl cinnamate; (33) 9-oxo-10(E),12(Z)-octadecadienoic acid; (34) 9-oxo-10(E),12(E)-octadecadienoic acid.

**Figure 2:**
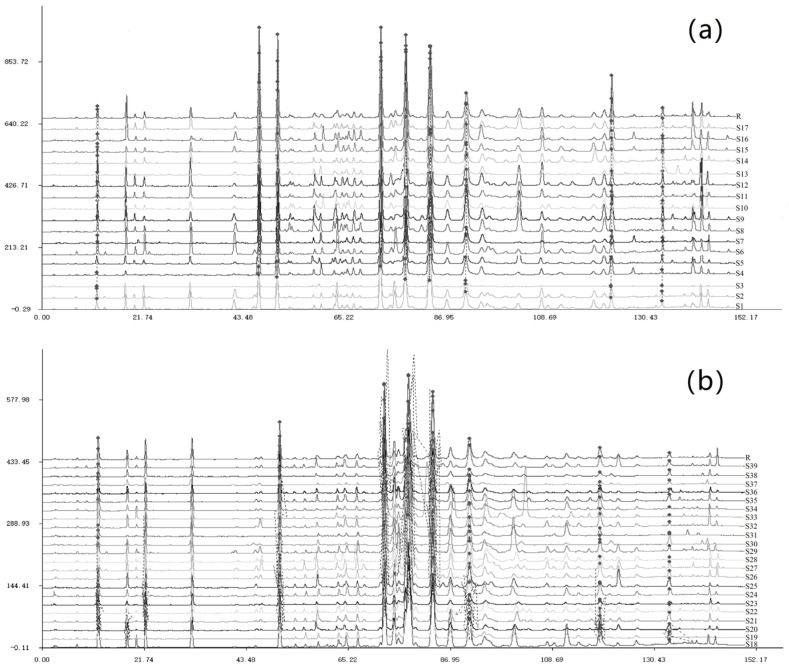
HPLC fingerprints of (**a**) 17 propolis samples S1 to S17 and (**b**) 22 propolis samples S18 to S39 from different provinces of China.

A HPLC profile of propolis sample S1 from Xinxiang apiary is demonstrated in Figure 1. As can be seen, the five obvious peaks were identified as 5-methoxy pinobanksin (peak 11), pinobanksin (peak 12), pinocembrin (peak 19), pinobanksin-3-acetate (peak 21), and chrysin (peak 22), respectively. The nearby *Populus* × *euramericana* cv. ‘Neva’ bud resins (B1) were also collected and analyzed, revealing the same five obvious peaks and phenolic composition (Figure 1 and Table 1). The content sum of five flavonoids in the propolis or bud resin constituted over 70% of the total phenolic content. Additionally, the similarity between propolis sample S1 and bud resin of *Populus* × *euramericana* cv. ‘Neva’ (B1) reached up to 0.933 (Supplement Table S3), indicating a strong similarity. The similar composition and high degree of similarity suggest that propolis sample S1 can originate from *Populus* × *euramericana* cv. ‘Neva’. Moreover, 16 propolis samples (S2 to S17) demonstrated HPLC profiles highly similar to S1 (Figure 2a and Supplement Figure S2), with a similarity degree ranging from 0.860 to 0.984 (Supplement Table S4). These 17 propolis samples shared a similar characteristic composition with high contents of five flavonoids. In total, 32 phenolic compounds were identified and quantified in these 17 samples, including 11 phenolic acids, 8 phenolic acid esters, and 13 flavonoids (Supplement Table S1). These propolis samples contained a higher average ratio of 76% for flavonoids and a lower average ratio of 24% for phenolic acids and their esters. This type of propolis also exhibited another notable characteristic—a lower content sum of 3,4-dimethoxy cinnamic acid and cinnamic acid—which accounted for only a quarter of the pinobanksin content, while the 5-methoxy pinobanksin content was more than half of the pinobanksin content. Furthermore, the chromatogram similarities between the 17 propolis samples (S1 to S17) and bud resin of *Populus* × *euramericana* cv. ‘Neva’ (B1) ranged from 0.768 to 0.933 (Supplement Table S3). The similarity average amounted to 0.891, a relatively high level. These results suggest that the botanical origin of these propolis samples may be *Populus* × *euramericana* cv. ‘Neva’.

### 3.2. Characteristics of 22 Propolis Samples and Bud Resin of Populus Simonii × P. nigra

Figure 1 displays the HPLC profile of propolis sample S18 from the Yichun apiary, which differs significantly from sample S1. The 32 compounds in sample S18 were identified, including four characteristic compounds: pinobanksin, pinocembrin, pinobanksin-3-acetate, and chrysin (Figure 1 and Table 1). These four compounds made up approximately half of the total phenolic content. To determine the botanical source of S18, we collected bud resins from several species of Populus trees (*Populus ussuriensis* Kom., *Populus koreana* Rehd., *Populus cathayana* Rehd., and *Populus Simonii* × *P*. *nigra*) in the vicinity of the apiary. A comparison between sample S18 and the bud resins of these four Populus species revealed that the chromatogram of *Populus Simonii* × *P*. *nigra* (B5) was highly similar to that of S18 (Figure 1), while the chromatogram of S18 noticeably differed from those of bud resins of *Populus ussuriensis* Kom. (B2), *Populus koreana* Rehd. (B3), and *Populus cathayana* Rehd. (B4). Additionally, the similarities between S18 and the bud resins of *Populus Simonii* × *P*. *nigra* (B5) were found to be 0.966 (Supplement Table S5). Furthermore, the flavonoids/phenolics ratios of the bud resin of *Populus Simonii* × *P*. *nigra* (B5) and sample S18 accounted for about 50%. These results suggest that the botanical origin of S18 propolis sample is likely *Populus Simonii* × *P*. *nigra*.

Upon comparing the chromatograms and phenolic compositions of the remaining propolis samples (S19 to S39), we found that they were highly similar with S18 (Figure 2b and Supplement Figure S3). The HPLC fingerprints exhibited eight major peaks, indicating a high degree of similarity. The similarities of samples (S18 to S39) ranged from 0.818 to 0.986 (Supplement Table S6). They shared the four characteristic flavonoids in their HPLC profiles as a distinctive feature. These propolis samples exhibited an average flavonoid/phenolic acid and ester ratio of 73% and 27%, respectively (Supplement Table S2). Another notable feature of these propolis samples was that the contents of 5-methoxy pinobanksin were lower half than those of pinobanksin. Additionally, we analyzed the chromatogram similarities between the 22 propolis samples (S18 to S39) and the bud resin of four Populus trees (B2 to B5) (Supplement Table S5). The similarities between the bud resin of *Populus Simonii* × *P*. *nigra* (B5) and the 22 propolis samples (S18 to S39) ranged from 0.606 to 0.975, with an average similarity of 0.910. These results show that the botanical origins of the 22 propolis samples are likely *Populus Simonii* × *P*. *nigra*.

### 3.3. Antioxidant Activities of 39 Propolis Samples

Table 2 illustrates the ORAC values of different extracts of 39 propolis samples using five different solvents. It is apparent that the ORAC values significantly varied with solvent types. Out of the 39 propolis samples tested, the ethanolic extracts using 75% ethanol showed the highest ORAC values, with an average of 444,144 μmol TE/100 g, which was significantly higher than the ORAC values of other extracts—approximately 1.4 times, 1.5 times, 37 times, and 42 times higher than those of the diethyl ether, ethyl acetate, petroleum ether, and water extracts, respectively. It is important to note that all 39 propolis ethanolic extracts had high levels of ORAC, with over one-third of the ORAC values above 500,000 μmol TE/100 g. The highest ORAC value recorded was 842,096 μmol TE/100 g (S8). Interestingly, the two groups of propolis samples showed similar average levels for the ORAC assay, with average ORAC values of 467,775 and 425,885 μmol TE/100 g for propolis ethanolic extracts.

To further determine the antioxidant activities of propolis samples, the scavenging capacity of the superoxide anion free radical was utilized. Based on the above results from the ORAC assay, 75% ethanol was used to extract the propolis samples as the solvent. Table 2 shows the IC_50_ values for scavenging the superoxide anion free radical for 39 propolis ethanolic extracts. The results indicate that approximately 70% of the propolis samples had an IC_50_ value of less than 1.00 mg/mL. The propolis sample S18 was the lowest IC_50_ value with a value of 0.30 mg/mL, which is comparable to the positive control Trolox. Furthermore, two groups of propolis samples exhibited similar scavenging capacities for the superoxide anion free radical, with their IC_50_ averages being 0.80 and 0.95 mg/mL, respectively.

## 4. Discussion

China is the leading global producer of propolis, and it is believed that Populus species are the primary sources of Chinese propolis [5,6,14]. However, the precise Populus species responsible for Chinese propolis production remained unknown until recently. In our previous study, we collected 98 propolis samples from 21 provinces in China [15] and categorized them into different types based on their compound characteristics. Our previous research discovered that the first type of propolis originates from *Populus canadensis* Moench, comprising 25 propolis samples [15]. For the remaining propolis samples, among the 98 samples, the present research revealed that 17 propolis samples and the bud resin of *Populus* × *euramericana* cv. ‘Neva’ shared the same five predominant flavonoids (5-methoxy pinobanksin, pinobanksin, pinocembrin, pinobanksin-3-acetate, and chrysin). Similarly, 22 propolis samples and the bud resin of *Populus Simonii* × *P*. *nigra* shared the same four predominant flavonoids (pinobanksin, pinocembrin, pinobanksin-3-acetate and chrysin). Moreover, the HPLC chromatograms of these propolis samples and bud resins had very high similarities (Figure 1 and Figure 2, Supplement Figures S2 and S3, Supplement Tables S1 and S2), respectively. Therefore, we conclude that 17 propolis samples (from S1 to S17) can be classified as the second type of propolis originated from *Populus* × *euramericana* cv. ‘Neva’; 22 propolis samples (from S18 to S39) can be classified as the third type of propolis from *Populus Simonii* × *P*. *nigra*. In future work, we will focus on the plant origins of the remaining 34 propolis samples.

In ancient China, propolis was never mentioned in pharmacopeias due to the non-collection of propolis for native honey bees, *Apis cerana* L. It was not until the introduction of the western honeybee, *Apis mellifera* L., in 1896 that propolis production and usage began to grow [15]. In Europe, *Populus nigra* L. was the primary source of resins collected by western honeybees. However, due to the rarity of this poplar species in China, it is likely that western honeybees in China collected resins from other plants to adapt to their new environment. Our previous research has already identified *Populus canadensis* Moench as the source of the first type of Chinese propolis [15], which is a hybrid of *Populus nigra* L. and *Populus deltoides* L. [16]. In this study, our results identified the botanical origins of the second and third types of Chinese propolis, from *Populus* × *euramericana* cv. ‘Neva’, a hybrid variety species of *Populus nigra* L. × *P*. *deltoids* L.; and *Populus Simonii* × *P*. *nigra*, a hybrid variety of *Populus nigra* L. and *Populus simonii*. Consequently, we speculate that the plant origins of Chinese propolis can be traced back to *Populus nigra* L. (a hybrid poplar). Several Populus species worldwide have been identified as sources of popular propolis, including *Populus suaveolens* [17], *Populus italica* [18], *Populus tremuloides* [19], *Populus nigra* L. [8], *Populus alba* [20], *Populus pyramidalis* [21], and *Populus fremontii*. Among these, *Populus italica*, *Populus nigra* L., and *Populus fremontii* [22] belong to the *Aigeiros Duby* section, while *Populus alba*, *Populus fremontii*, and *Populus pyramidalis* are included in the *Leuce* Duby section, and *Populus suaveolens* is part of the *Tacamahaca* Spach section. This study can be the first to identify *Populus* × *euramericana* cv. ‘Neva’ and *Populus Simonii* × *P*. *nigra* as the botanical origins of propolis.

Numerous studies have shown that oxidative damage caused by free radicals or oxidative stress in the body is closely related to various degenerative diseases [13]. Consequently, interest in evaluating the antioxidant capacity of natural supplements and foods for promoting health and preventing diseases has risen. In this study, we assessed the antioxidant properties of two types of poplar-derived propolis samples. Our findings revealed that these propolis samples exhibited excellent antioxidant activities, as shown by their ORAC and superoxide anion free radical scavenging capacity. Among the solvents utilized for extraction, 75% ethanol was found to be the most efficient in extracting phenolic compounds compared to diethyl ether, ethyl acetate, petroleum ether, and water. A similar result was observed by Silva [23], reporting that the ORAC values of 75% ethanolic extracts of propolis from the southern region of Uruguay (poplar-tree origin) ranged from 1.8 to 9.0 μmol TE/mg. The US Department of Agriculture Website provides a useful list of ORAC values of vegetable and fruit extracts (http://www.ars.usda.gov/SP2UserFiles/Place/12354500/Data/ORAC/ORAC_R2.pdf, accessed on 10 December 2022). Our propolis samples had an average ORAC value of 444,144 μmol TE/100 g, which is remarkably high—approximately four times higher than acai, 30 times higher than ginger root, 45 times higher than blueberries, 100 times higher than red wine, 180 times higher than cabbage, and 300 times higher than honey. For superoxide anion free radical scavenging capacity assay, the 75% ethanolic extracts of these two types of propolis samples exhibited similar and excellent radical scavenging activity.

Our results indicate that the ethanolic extract of propolis possesses excellent antioxidant properties. Furthermore, a positive correlation was observed between the antioxidant activity and the total flavonoid content of propolis samples, such as S8, S12, S16, S27, S18, and S29. Based on the high flavonoid content in these propolis samples, these two types of propolis can be categorized as flavonoid-rich propolis. The flavonoid contents in these samples were significantly higher than those of phenolic acid and their esters, with flavonoids comprising over 65% of total phenolics on average (Supplement Table S1 and S2). As such, flavonoids seem to endow these two types of poplar-type propolis with remarkable antioxidant activities [24,25,26]. Furthermore, it is worth mentioning that propolis possesses important pharmacological effects; however, it can cause allergic eczematous contact dermatitis and an allergic predisposition [27,28,29]. It is well-known that the main allergens of propolis are some caffeic acid derivatives, for example, phenylmethyl caffeate, benzyl cinnamate, etc. [30,31]. Given the low phenolic acid and ester ratio, these flavonoid-rich propolis can be utilized to create low-allergen nutraceuticals.

## 5. Conclusions

Our results reveal that the botanical origins of two types of poplar-type propolis samples are *Populus* × *euramericana* cv. ‘Neva’ and *Populus Simonii* × *P*. *nigra*, respectively. We also report here for the first time that these two types of poplar-type propolis samples can be characterized by five (5-methoxy pinobanksin, pinobanksin, pinocembrin, pinobanksin-3-acetate, and chrysin) or four (pinobanksin, pinocembrin, pinobanksin-3-acetate, and chrysin) predominant flavonoids, with the average characteristic flavonoid contents being more than two-thirds of that of the total phenolics. These two types of propolis can be recognized as flavonoid-rich propolis. Additionally, the 75% ethanolic extracts of these propolis samples exhibit stronger antioxidant activities than most commonly consumed natural foods, which can be attributed to their high flavonoid content. These findings provide reliable evidence for utilizing these two types of propolis to develop novel low-allergen and high-antioxidant nutraceuticals.

## Figures and Tables

**Table 1:** Phenolic contents of propolis and poplar bud resin samples (mg/g propolis).

Peak Number	Components	S1	S18	B1	B2	B3	B4	B5
1	3,4 Dihydroxybenzaldehyde	—	0.21	0.20	—	—	0.54	0.89
2	Caffeic acid	1.74	7.37	—	0.96	—	0.79	7.62
3	Vanillin	—	0.16	0.41	0.02	—	—	0.13
4	*p*-Coumaric acid	1.10	0.96	—	0.05	—	0.10	0.30
5	Ferulic acid	0.83	1.89	—	0.11	—	0.13	2.50
6	Isoferulic acid	0.77	8.73	1.05	0.02	—	0.17	7.57
7	Benzoic acid	—	2.05	—	—	—	—	—
8	3,4-Dimethoxy cinnamic acid	1.16	5.29	—	—	—	0.59	4.23
9	Cinnamic acid	0.12	—	—	—	—	—	0.03
10	4-Methoxy cinnamic acid	—	0.36	—	—	—	—	0.23
11	5-Methoxy pinobanksin	21.60	—	9.97	—	—	—	0.43
12	Pinobanksin	15.20	13.11	7.35	4.43	9.54	0.75	5.67
13	Quercetin	0.61	—	0.29	0.04	—	—	0.25
14	Alpinetin	0.81	—	0.53	0.69	—	—	0.20
15	Kaempferol	0.66	0.86	0.40	0.29	—	—	0.18
16	Cinnamylideneacetic acid	1.25	0.68	—	—	—	—	—
17	Apigenin	1.17	2.45	—	—	—	—	0.45
18	Isorhamnetin	2.04	—	1.78	—	—	—	—
19	Pinocembrin	21.64	29.56	10.41	17.75	59.35	3.24	15.43
20	Benzyl caffeate	5.28	33.03	0.08	4.82	11.43	4.56	14.94
21	Pinobanksin-3-acetate	34.71	52.50	22.13	24.42	58.32	8.26	16.66
22	Chrysin	27.34	38.84	7.93	10.41	22.77	6.17	7.04
23	Phenethyl caffeate	6.46	27.53	0.06	4.23	2.90	0.91	23.11
24	Galangin	14.58	14.05	6.19	5.94	10.75	0.81	5.56
25	Benzyl *p*-coumarate	4.31	—	2.07	0.56	1.70	—	1.96
26	Benzyl ferulate	—	—	—	1.35	—	—	3.53
27	Cinnamyl caffeate	—	—	0.79	0.65	1.83	—	2.98
28	Pinostrobin	1.09	9.98	—	1.28	2.29	—	6.70
29	Tectochrysin	1.66	11.96	0.12	0.99	1.99	0.72	1.10
30	Cinnamyl *p*-cinnamate	—	—	1.29	0.51	2.00	—	—
31	Cinnamyl cinnamate	0.66	—	—	—	—	—	—
32	4-methoxy cinnamyl cinnamate	—	—	0.72	—	—	—	—
SFC		143.11	173.31	67.10	66.24	165.01	19.95	59.67
SPC		166.79	261.57	73.77	79.52	184.87	27.74	129.69

**Note:** “—”: No detected. S1 refers to Henan, Xinxiang propolis; S18 refers to Heilongjiang, Yichun propolis; B1, B2, B3, B4, B5 refer to bud resin of *Populus* × *euramericana* cv. ‘Neva’, *Populus ussuriensis* Kom., *Populus koreana* Rehd., *Populus cathayana* Rehd., and *Populus Simonii* × *P*. *nigra*, SFC represents the sum of flavonoid contents, SPC represents the sum of phenolic contents.

**Table 2:** The oxygen radical absorbance capacity (ORAC) values and the half inhibition concentrations (IC_50_) for scavenging of superoxide anion free radical determined for 39 propolis extracts.

Samples	ORAC (μmol TE/100 g Propolis)					Scavenging of Superoxide Anion Free Radical IC_50_ (mg/mL)
	75% Ethanolic Extract	Water Extract	Diethyl Ether Extract	Ethyl Acetate Extract	Petroleum Ether Extract	75% Ethanolic Extract
S1	379,174 ± 38,346 ^b^	11,805 ± 1244 ^c^	541,017 ± 38,270 ^a^	354,408 ± 60,892 ^b^	15,967 ± 1030 ^c^	0.95 ± 0.05
S2	637,888 ± 30,346 ^a^	12 586 ± 442 ^d^	288,927 ± 27,844 ^c^	401,763 ± 17,416 ^b^	2678 ± 387 ^d^	0.43 ± 0.02
S3	503,720 ± 39,630 ^a^	13,240 ± 159 ^d^	216,427 ± 1160 ^c^	401,750 ± 23,323 ^b^	11,147 ± 912 ^d^	0.48 ± 0.02
S4	154,320 ± 20,879 ^c^	10,471 ± 1063 ^d^	403,728 ± 57,412 ^b^	678,206 ± 60,995 ^a^	6202 ± 1091 ^d^	1.75 ± 0.09
S5	196,190 ± 13,218 ^c^	7178 ± 448 ^d^	463,542 ± 17,734 ^b^	578,997 ± 17,576 ^a^	6081 ± 388 ^d^	1.71 ± 0.07
S6	441,886 ± 21,764 ^a^	16,097 ± 430 ^d^	358,347 ± 18,360 ^b^	293,998 ± 20,864 ^c^	8328 ± 999 ^d^	0.59 ± 0.03
S7	187,915 ± 21,072 ^b^	11,874 ± 762 ^d^	257,527 ± 18,609 ^a^	118,896 ± 8748 ^c^	19,291 ± 2113 ^d^	1.73 ± 0.09
S8	842,096 ± 54,099 ^a^	14,168 ± 888 ^d^	401,668 ± 40,459 ^b^	305,723 ± 27,879 ^c^	7754 ± 1155 ^d^	0.39 ± 0.02
S9	567,422 ± 34,823 ^a^	9416 ± 611 ^d^	390,051 ± 9873 ^b^	271,492 ± 32,091 ^c^	36,559 ± 3499 ^d^	0.55 ± 0.02
S10	606,311 ± 57,166 ^a^	7257 ± 640 ^d^	180,752 ± 12,069 ^b^	122,861 ± 19,821 ^c^	12,077 ± 743 ^d^	0.53 ± 0.02
S11	286,677 ± 76222 ^a^	9050 ± 171 ^c^	188,670 ± 3804 ^b^	197,223 ± 3884 ^b^	10,317 ± 973 ^c^	1.19 ± 0.05
S12	766,707 ± 35473 ^a^	12,786 ± 562 ^d^	134,678 ± 9909 ^b^	80,188 ± 7718 ^c^	21,753 ± 2131 ^d^	0.30 ± 0.01
S13	458,610 ± 24,788 ^a^	6917 ± 212 ^c^	142,825 ± 8659 ^b^	127,059 ± 30,766 ^b^	5453 ± 15 ^c^	0.71 ± 0.03
S14	303,801 ± 24,541 ^a^	15,246 ± 541 ^d^	131,981 ± 5194 ^c^	229,725 ± 14,465 ^b^	5615 ± 117 ^d^	0.77 ± 0.03
S15	476,522 ± 10496 ^a^	9668 ± 79 ^c^	211,215 ± 25,410 ^b^	232,973 ± 14,871 ^b^	5302 ± 18 ^c^	0.58 ± 0.03
S16	675,597 ± 30,595 ^a^	10,694 ± 735 ^c^	293,749 ± 30,868 ^b^	263,938 ± 9027 ^b^	13,550 ± 3014 ^c^	0.36 ± 0.02
S17	467,337 ± 29,400 ^b^	9699 ± 595 ^d^	526,158 ± 11,157 ^a^	419,262 ± 32,815 ^c^	8842 ± 97 ^d^	0.50 ± 0.03
S18	736,339 ± 35,647 ^a^	11,908 ± 877 ^d^	271,950 ± 30,127 ^c^	497,525 ± 40,145 ^b^	49,493 ± 2424 ^d^	0.36 ± 0.02
S19	482,379 ± 24,788 ^a^	12,522 ± 56 ^d^	196,733 ± 8501 ^c^	294,190 ± 8554 ^b^	6297 ± 485 ^d^	0.76 ± 0.03
S20	281,680 ± 26,996 ^c^	9732 ± 635 ^d^	390,055 ± 20,515 ^b^	603,396 ± 5730 ^a^	12,625 ± 597 ^d^	1.39 ± 0.05
S21	470,017 ± 20,211 ^a^	15,517 ± 896 ^d^	248,603 ± 7834 ^b^	142,056 ± 32,729 ^c^	5687 ± 317 ^d^	0.79 ± 0.02
S22	615,606 ± 22,180 ^a^	12,118 ± 563 ^c^	304,615 ± 17,192 ^b^	299,233 ± 526 ^b^	3571 ± 420 ^c^	0.69 ± 0.04
S23	246,492 ± 12,874 ^a^	6813 ± 183 ^c^	220,463 ± 33,587 ^a^	136,812 ± 2188 ^b^	3963 ± 176 ^c^	1.47 ± 0.04
S24	563,044 ± 42,410 ^a^	10,160 ± 445 ^c^	430,327 ± 31,162 ^b^	592,005 ± 43,569 ^a^	7484 ± 642 ^c^	0.56 ± 0.02
S25	444,347 ± 16,816 ^b^	12,208 ± 626 ^c^	487,242 ± 38,124 ^a^	457,150 ± 3608 ^ab^	12,495 ± 2698 ^c^	0.77 ± 0.03
S26	508,994 ± 15,894 ^a^	7747 ± 499 ^d^	384,567 ± 18,935 ^b^	223,639 ± 12,063 ^c^	6250 ± 318 ^d^	0.73 ± 0.03
S27	741,365 ± 10,705 ^a^	15,542 ± 245 ^d^	315,533 ± 16,578 ^b^	223,889 ± 10,257 ^c^	1292 ± 379 ^d^	0.52 ± 0.03
S28	508,984 ± 17,947 ^a^	14,611 ± 652 ^c^	515,912 ± 46,377 ^a^	412,457 ± 25,706 ^b^	10,631 ± 857 ^c^	0.74 ± 0.04
S29	638,041 ± 37,772 ^a^	14,904 ± 888 ^d^	281,913 ± 44,162 ^b^	185,664 ± 77,591 ^c^	2060 ± 794 ^d^	0.61 ± 0.02
S30	275,051 ± 5852 ^b^	8396 ± 359 ^d^	79,672 ± 2888 ^c^	553,287 ± 15,235 ^a^	6626 ± 605 ^d^	1.19 ± 0.03
S31	263,353 ± 14,028 ^a^	8876 ± 648 ^c^	94,173 ± 59,632 ^b^	93,398 ± 9914 ^b^	4745 ± 13 ^c^	1.49 ± 0.03
S32	434,623 ± 21,008 ^c^	8562 ± 484 ^d^	756,597 ± 9006 ^a^	589,004 ± 26,476 ^b^	12,390 ± 985 ^d^	0.84 ± 0.05
S33	417,722 ± 9763 ^a^	6040 ± 1249 ^c^	129,200 ± 15,595 ^b^	124,519 ± 7174 ^b^	8321 ± 865 ^c^	0.79 ± 0.02
S34	298,776 ± 10,462 ^a^	12,513 ± 136 ^c^	176,045 ± 24,409 ^b^	159,947 ± 1005 ^b^	7862 ± 361 ^c^	0.86 ± 0.04
S35	450,131 ± 68,499 ^a^	9297 ± 518 ^c^	301,762 ± 33,018 ^b^	257,184 ± 40,635 ^b^	5744 ± 320 ^c^	0.69 ± 0.03
S36	261,021 ± 15,046 ^b^	8557 ± 783 ^e^	330,817 ± 32,361 ^a^	203,338 ± 24,367 ^c^	77,358 ± 6155 ^d^	1.68 ± 0.07
S37	108,401 ± 22,155 ^b^	9680 ± 605 ^c^	142,151 ± 23,391 ^b^	257,228 ± 27,749 ^a^	6588 ± 160 ^c^	1.83 ± 0.08
S38	284,170 ± 10,543 ^a^	3021 ± 183 ^d^	85,214 ± 5677 ^b^	81,445 ± 2061 ^b^	23,686 ± 5085 ^c^	1.13 ± 0.06
S39	338,923 ± 20,220 ^b^	7466 ± 316 ^d^	259,514 ± 8095 ^c^	558,601 ± 23,121 ^a^	6423 ± 665 ^d^	1.02 ± 0.05

**Note:** Results are shown as the mean ± SD (*n* = 3); Values with superscript letters a, b, c, d, and e are significantly different across columns (*p* < 0.05).

## Data Availability

The data used to support the findings of this study can be made available by the corresponding author upon request.

## Supplementary Materials

The following supporting information can be downloaded at: https://www.mdpi.com/article/10.3390/foods12122304/s1, Figure S1: The sites of the collection of 39 propolis and 5 poplar bud resin samples from different provinces of China; Figure S2: HPLC chromatograms of 16 propolis samples (S2 to S17) from different provinces of China; Figure S3: HPLC chromatograms of 21 propolis samples (S19 to S39) from different provinces of China; Table S1: Phenolic contents of propolis and bud resin of *Populus* × *euramericana* cv. ‘Neva’; Table S2: Phenolic contents of propolis and four poplar bud resin samples; Table S3: Similarities of 17 propolis samples and bud resin of *Populus* × *euramericana* cv. ‘Neva’; Table S4: Similarity evaluation of 17 propolis samples; Table S5: Similarities of 22 propolis and 4 poplar bud resin samples; Table S6: Similarity evaluation of 22 propolis samples.
