# Supplementary material for: Botanical Origins and Antioxidant Activities of Two Types of Flavonoid-Rich Poplar-Type Propolis
Source: Foods. 2023 Jun 7;12(12):2304. doi: 10.3390/foods12122304 (PMC10297319; doi:10.3390/foods12122304)
Supplement: Supplementary file 1 [file foods-12-02304-s001.zip › foods-2427377-supplementary.pdf]

Figure S1

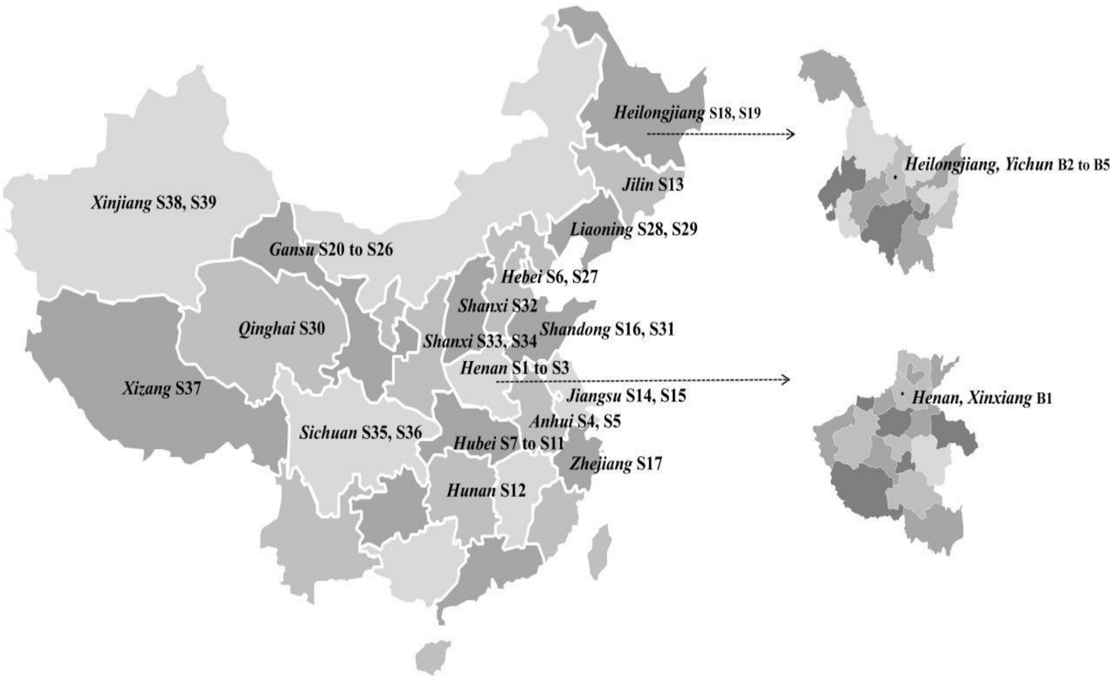

Figure S1. The sites of the collection of 39 propolis and 5 poplar bud resin samples from different provinces of China.

Figure S2

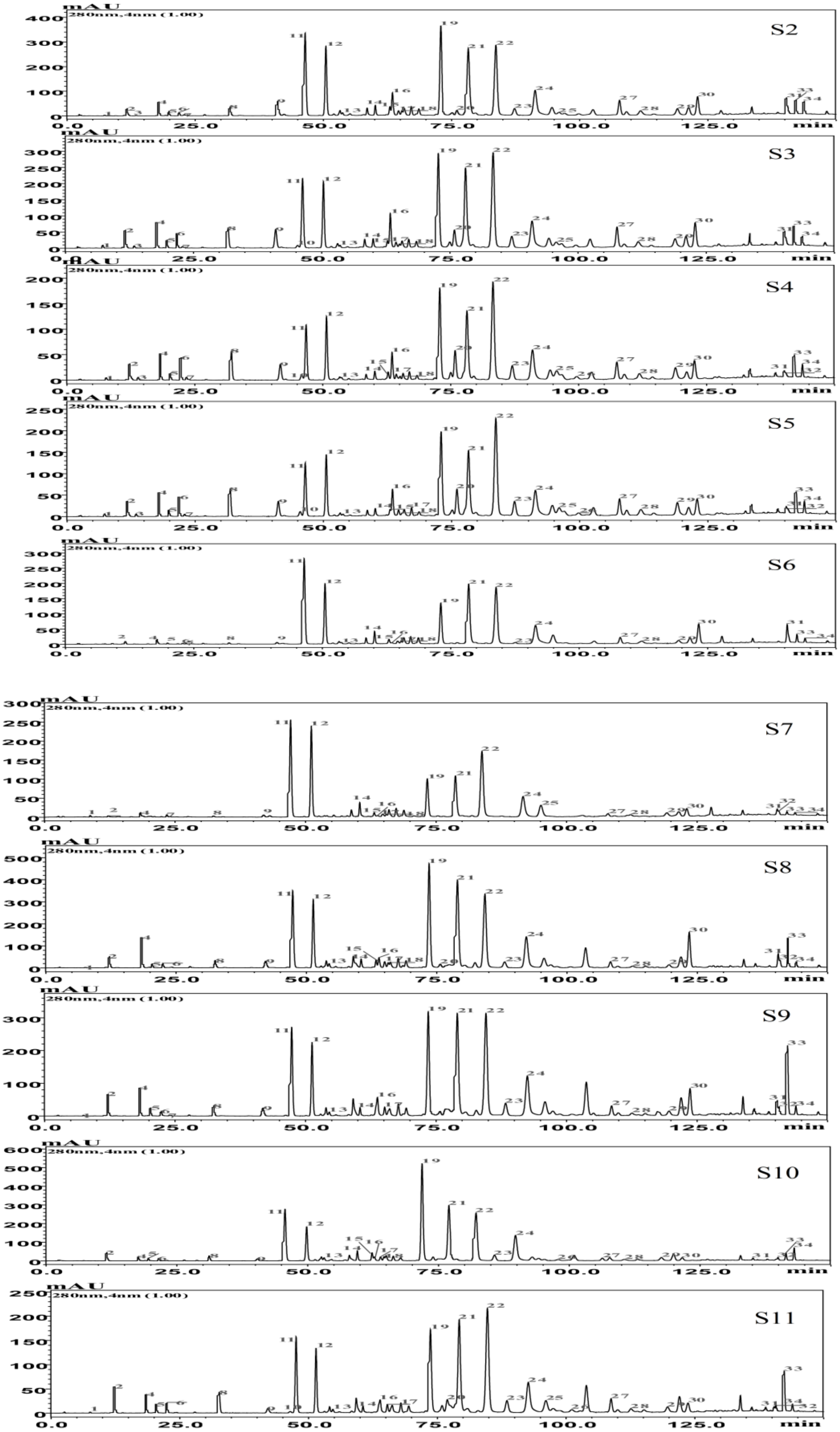

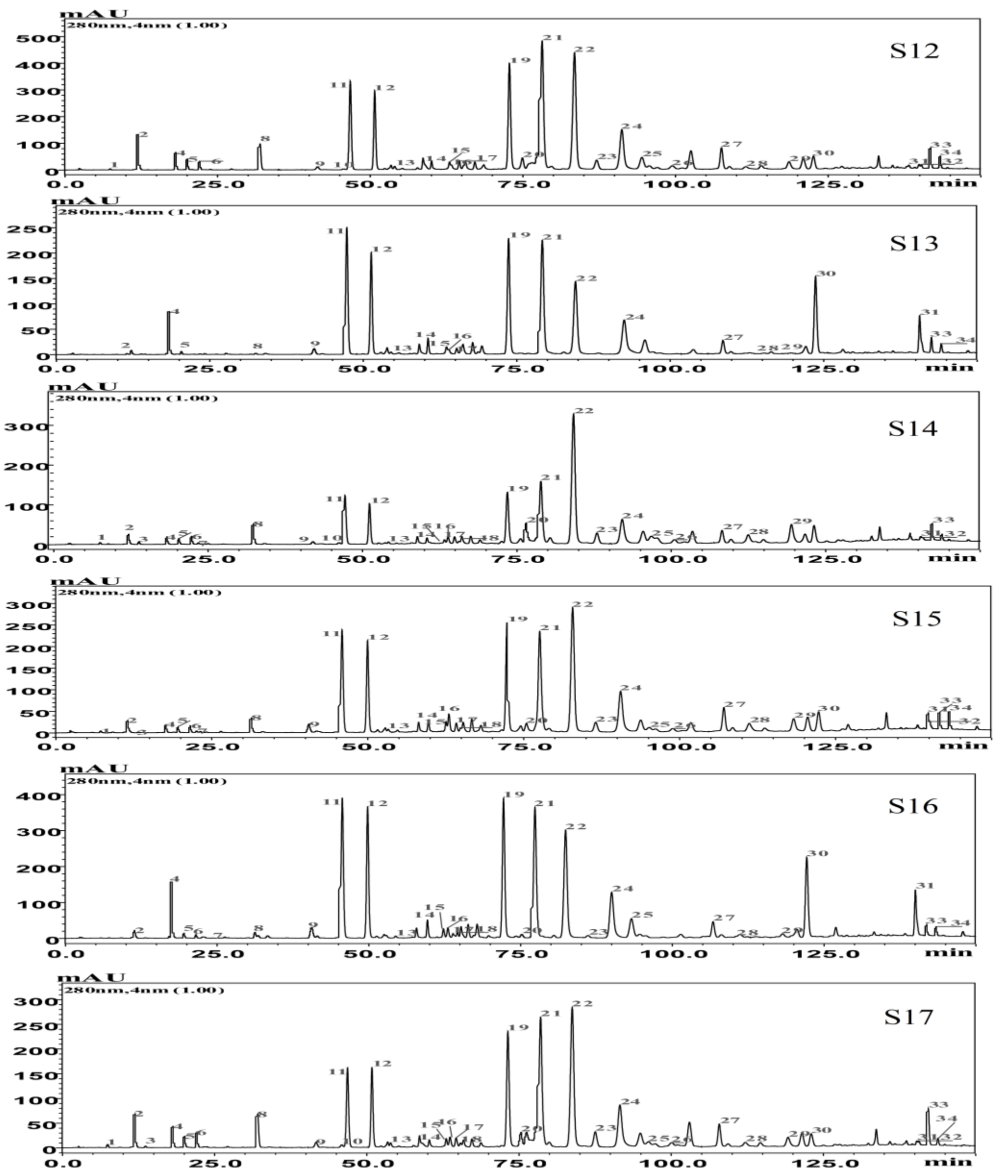

Figure S2. HPLC chromatograms of 16 propolis sample S2 to S17 from different provinces of China

Note: (1) 3,4-Dihydroxybenzyl aldehyde; (2) Caffeic acid; (3) Vanillin; (4) *p*-Coumaric acid; (5) Ferulic acid; (6) Isoferulic acid; (7) Benzoic acid; (8) 3, 4-Dimethoxy cinnamic acid; (9) Cinnamic acid; (10) 4-Methoxy cinnamic acid; (11) 5-Methoxy pinobanksin; (12) Pinobanksin; (13) Quercetin; (14) Alpinetin; (15) Kaempferol; (16) Cinnamylideactic acid; (17) Apigenin; (18) Isorhamnetin; (19) Pinocembrin; (20) Benzyl Caffeate; (21) Pinobanksin-3-acetate; (22) Chrysin; (23) Phenethyl caffeate; (24) Galangin; (25) Benzyl *p*-coumarate; (26) Benzyl ferulate; (27) Cinnamyl caffeate; (28) Pinostrobin; (29) Tectochrysin; (30) Cinnamyl *p*-cinnamate; (31) Cinnamyl cinnamate; (32) 4-Methoxy cinnamyl cinnamate; (33) 9-oxo-10(E),12(Z)-octadecadienoic acid; (34) 9-oxo-10(E),12(E)-octadecadienoic acid.

Figure S3

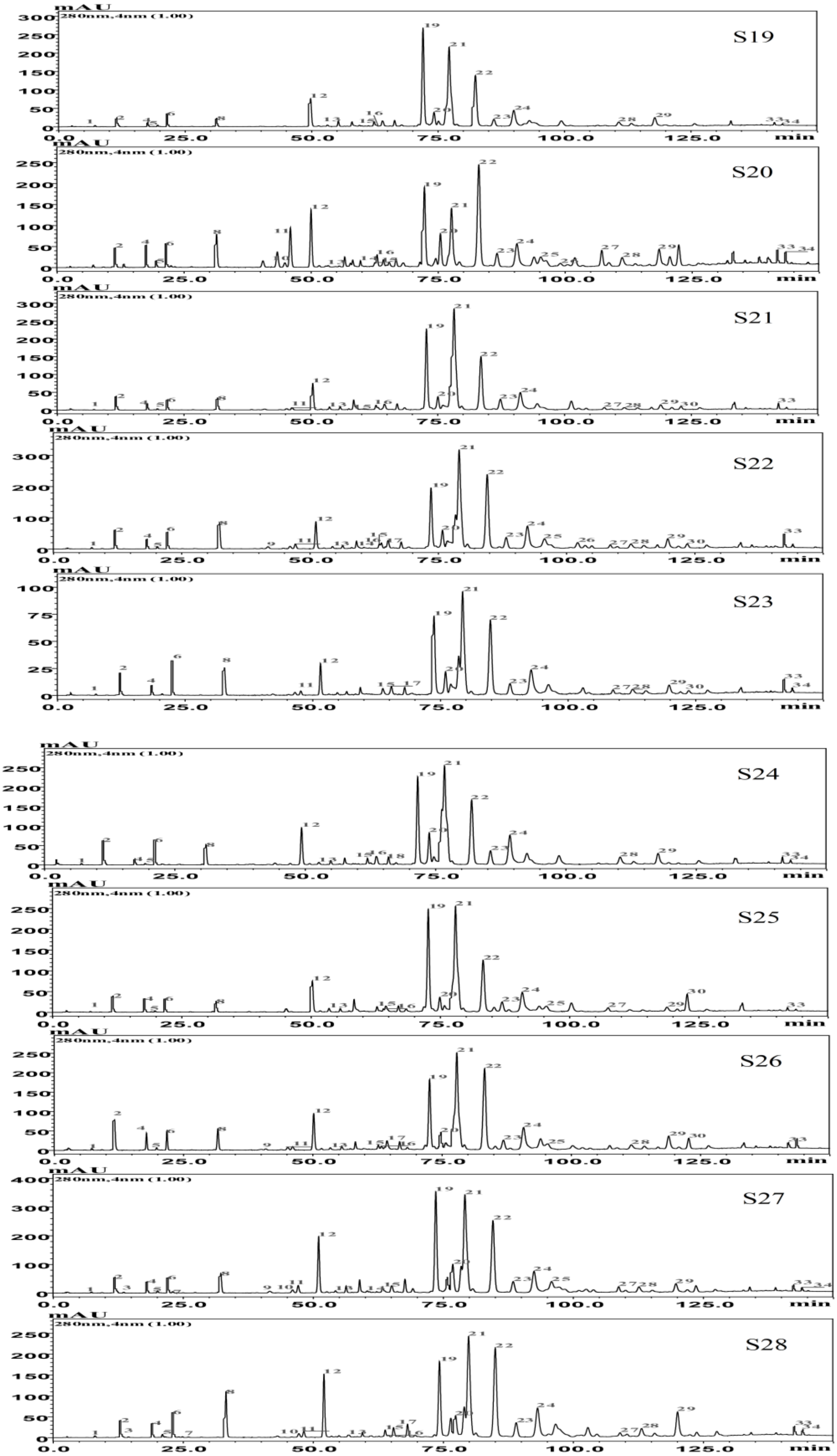
mAU
280nm,4nm (1.00)
S19
S20
S21
S22
S23
S24
S25
S26
S27
S28
0.0
25.0
50.0
75.0
100.0
125.0
min

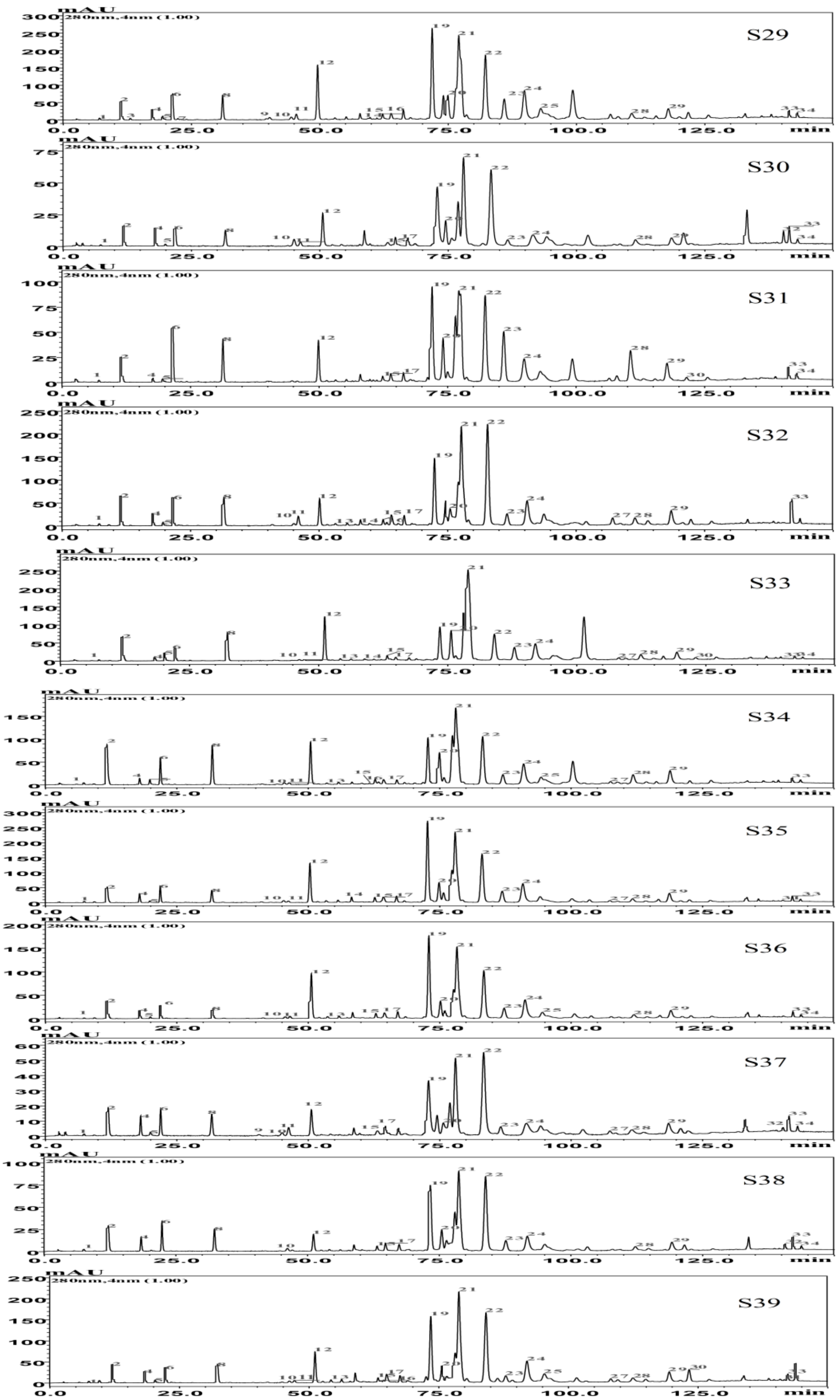

Figure S3. HPLC chromatograms of 21 propolis samples S19 to S39 from different provinces of China.

Note: (1) 3,4-Dihydroxybenzyl aldehyde; (2) Caffeic acid; (3) Vanillin; (4) *p*-Coumaric acid; (5)

Ferulic acid; (6) Isoferulic acid; (7) Benzoic acid; (8) 3, 4-Dimethoxy cinnamic acid; (9) Cinnamic acid; (10) 4-Methoxy cinnamic acid; (11) 5-Methoxy pinobanksin; (12) Pinobanksin; (13) Quercetin; (14) Alpinetin; (15) Kaempferol; (16) Cinnamylideactic acid; (17) Apigenin; (18) Isorhamnetin; (19) Pinocembrin; (20) Benzyl Caffeate; (21) Pinobanksin-3-acetate; (22) Chrysin; (23) Phenethyl caffeate; (24) Galangin; (25) Benzyl *p*-coumarate; (26) Benzyl ferulate; (27) Cinnamyl caffeate; (28) Pinostrobin; (29) Tectochrysin; (30) Cinnamyl *p*-cinnamate; (31) Cinnamyl cinnamate; (32) 4-Methoxy cinnamyl cinnamate; (33) 9-oxo-10(E),12(Z)-octadecadienoic acid; (34) 9-oxo-10(E),12(E)-octadecadienoic acid.

Table S1. Phenolic contents of propolis and bud resin of *Populus* × *euramericana cv.* 'Neva' (mg/g propolis).

Peak Number	Components	S1	S2	S3	S4	S5	S6	S7	S8	S9	S10	S11	S12	S13	S14	S15	S16	S17	B1
1	3,4 Dihydroxybenzaldehyde	—	0.08	0.26	0.16	0.19	—	0.12	0.07	0.07	—	0.07	0.12	—	0.14	0.12	—	0.19	0.20
2	Caffeic acid	1.74	1.61	2.94	1.75	1.95	0.45	0.18	2.71	3.20	2.28	2.58	6.52	0.46	1.33	1.58	1.28	3.49	—
3	Vanillin	—	0.20	0.43	0.37	0.40	—	—	—	—	—	—	—	—	0.38	0.19	—	0.25	0.41
4	*p*-Coumaric acid	1.10	1.84	2.62	1.77	1.85	0.55	0.36	4.80	2.85	0.77	1.25	2.07	2.89	0.66	0.63	5.45	1.46	—
5	Ferulic acid	0.83	1.17	1.55	0.92	1.00	0.36	—	1.13	1.51	0.91	1.15	2.36	0.47	1.01	0.92	0.98	1.44	—
6	Isoferulic acid	0.77	0.81	2.66	2.60	2.65	0.19	—	1.25	0.76	0.83	1.26	1.85	—	1.23	0.96	0.59	1.88	1.05
7	Benzoic acid	—	2.02	3.20	3.51	3.15	1.99	3.62	—	1.70	—	—	—	—	2.50	2.35	2.12	—	—
8	3,4-Dimethoxy cinnamic acid	1.16	1.51	2.53	2.39	2.63	0.20	0.15	1.43	1.29	1.10	1.77	3.99	0.10	2.15	1.40	0.70	2.81	—
9	Cinnamic acid	0.12	0.96	0.93	0.51	0.57	0.08	0.08	0.50	0.42	0.16	0.16	0.18	0.19	0.10	0.30	0.54	0.17	—
10	4-Methoxy cinnamic acid	—	—	0.57	0.76	0.81	—	—	—	—	—	0.24	0.24	—	0.28	—	—	0.40	—
11	5-Methoxy pinobanksin	21.60	41.45	26.99	13.23	15.49	34.89	30.67	41.35	31.69	34.83	18.20	39.54	30.00	14.71	29.63	49.47	19.60	9.97
12	Pinobanksin	15.20	30.71	22.45	13.30	15.72	21.76	25.21	32.05	23.05	19.81	13.58	30.49	21.04	10.67	22.99	40.84	17.12	7.35
13	Quercetin	0.61	0.78	0.54	0.32	0.34	0.33	—	1.21	0.78	1.08	0.49	0.84	0.30	0.31	0.49	0.38	0.63	0.29
14	Alpinetin	0.81	1.80	1.18	0.71	0.76	1.72	1.64	1.69	1.08	2.21	0.47	1.40	1.26	0.61	1.20	2.07	0.65	0.53
15	Kaempferol	0.66	1.37	0.81	0.63	0.49	0.60	0.38	1.34	—	1.58	—	1.11	0.58	0.33	0.90	0.90	0.60	0.40
16	Cinnamylideneacetic acid	1.25	4.99	5.65	2.86	3.39	0.28	0.29	2.60	4.09	1.12	1.95	0.86	0.54	1.01	2.31	1.49	1.14	—
17	Apigenin	1.17	1.46	1.32	0.85	0.88	0.73	0.57	2.12	1.66	1.43	1.16	2.13	0.78	1.23	1.48	0.85	1.34	—
18	Isorhamnetin	2.04	3.50	2.19	1.36	1.24	1.84	1.33	4.24	—	3.72	—	—	—	1.13	1.77	2.68	1.58	1.78
19	Pinocembrin	21.64	36.30	29.02	17.80	19.67	13.33	10.04	47.82	31.89	49.97	17.52	39.03	23.17	13.31	24.80	37.56	23.36	10.41
20	Benzyl caffeate	5.28	7.29	17.21	17.01	18.50	—	—	6.23	—	—	14.60	13.31	—	14.53	7.13	4.82	8.86	0.08
21	Pinobanksin-3-acetate	34.71	52.22	47.75	25.54	29.83	35.28	18.83	73.14	54.74	51.87	38.39	85.31	41.12	28.62	44.32	62.60	49.29	22.13
22	Chrysin	27.34	37.31	38.49	24.97	30.16	24.87	22.92	44.04	42.25	32.56	29.12	56.71	19.70	42.95	37.00	38.26	36.88	7.93
23	Phenethyl caffeate	6.46	8.56	10.71	8.38	10.40	0.91	—	8.12	11.87	8.96	7.74	10.83	—	8.20	6.88	1.73	9.52	0.06
24	Galangin	14.58	20.93	17.28	11.88	12.79	13.58	10.90	27.66	24.64	25.83	13.26	28.96	14.63	12.52	18.68	24.50	17.09	6.19

25	Benzyl *p*-coumarate	4.31	1.62	2.71	1.33	1.36	—	4.99	—	—	—	3.65	7.60	—	0.86	0.59	8.57	0.77	2.07
26	Benzyl ferulate	—	5.03	—	4.90	4.96	—	—	—	—	4.89	4.94	7.59	—	6.17	5.63	—	5.39	—
27	Cinnamyl caffeate	—	12.81	13.26	8.70	9.47	6.63	4.52	7.51	7.78	5.55	7.60	15.57	7.41	8.33	11.95	10.28	10.52	0.79
28	Pinostrobin	1.09	3.73	3.39	1.96	2.81	1.95	0.84	1.32	1.54	1.49	1.51	1.92	0.57	3.57	3.84	0.80	1.61	—
29	Tectochrysin	1.66	3.65	3.66	3.18	4.11	1.63	1.56	2.05	1.99	2.40	1.67	3.78	0.56	6.16	4.09	1.56	2.76	0.12
30	Cinnamyl *p*-cinnamate	—	10.29	10.58	6.48	6.88	9.18	4.80	18.25	10.73	4.20	4.76	7.96	17.21	—	7.68	25.00	5.54	1.29
31	Cinnamyl cinnamate	0.66	2.91	2.05	0.56	0.67	2.56	0.64	2.20	1.55	0.46	0.42	0.62	3.07	0.43	1.79	5.28	0.38	—
32	4-methoxy cinnamyl cinnamate	—	—	—	2.57	2.72	—	2.68	3.98	3.61	3.09	2.86	3.02	—	2.54	2.10	—	2.67	0.72
	SFC	143.11	235.21	195.07	115.73	134.29	152.51	124.89	280.03	215.31	228.78	135.37	291.22	153.71	136.12	191.19	262.47	172.51	67.10
	SPC	166.79	298.91	274.93	183.26	207.84	175.89	147.32	340.81	266.74	263.10	192.37	375.91	186.05	187.97	245.70	331.30	229.39	73.77

Note: "—": No detected. S1-S3 refer to Henan propolis; S4, S5 refer to Anhui propolis; S6 refers to Hebei propolis; S7-S11 refer to Hubei propolis; S12 refers to Hunan propolis; S13 refers to Jilin propolis; S14, S15 refer to Jiangsu propolis; S16 refers to Shandong propolis; S17 refers to Zhejiang propolis and B1 refer to bud resin of *Populus* × *euramericana* cv. 'Neva', SFC represents the sum of flavonoid contents, SPC represents the sum of phenolic contents.

Table S2. Phenolic contents of propolis and bud resins of four poplar (mg/g propolis).

Peak Number	Components	S18	S19	S20	S21	S22	S23	S24	S25	S26	S27	S28	S29	S30	S31	S32	S33	S34	S35	S36	S37	S38	S39	B2	B3	B4	B5
1	3,4 Dihydroxybenzaldehyde	0.21	0.11	—	0.06	0.13	0.02	0.11	0.06	0.14	0.11	0.14	0.16	0.04	0.07	0.14	0.09	0.12	0.13	0.06	0.05	0.07	0.12	—	—	0.54	0.89
2	Caffeic acid	7.37	1.41	1.62	1.98	3.09	1.02	3.32	2.01	4.17	3.02	2.15	2.89	0.93	1.45	3.46	3.85	4.85	2.87	2.16	1.06	1.53	2.30	0.96	—	0.79	7.62
3	Vanillin	0.16	—	—	—	—	—	—	—	—	0.25	0.18	0.32	—	—	—	—	—	—	—	—	—	—	0.02	—	—	0.13
4	*p*-Coumaric acid	0.96	0.40	0.65	0.56	1.07	0.30	0.53	1.08	1.44	1.33	1.18	1.03	0.52	0.14	0.95	0.40	0.47	1.02	0.61	0.47	0.57	0.97	0.05	—	0.10	0.30
5	Ferulic acid	1.89	0.35	0.28	0.32	0.54	—	0.31	0.28	0.46	0.57	0.55	0.75	0.23	0.31	0.57	1.42	0.76	0.43	0.32	0.31	—	0.51	0.11	—	0.13	2.50
6	Isoferulic acid	8.73	2.07	1.50	1.66	3.23	1.86	3.66	1.93	2.91	3.16	3.63	4.26	0.97	3.14	3.62	2.09	3.51	3.26	1.68	1.21	2.03	2.21	0.02	—	0.17	7.57
7	Benzoic acid	2.05	—	—	—	—	—	—	—	—	3.57	1.70	3.13	—	—	—	—	—	—	—	—	—	—	—	—	—	—
8	3,4-Dimethoxy cinnamic acid	5.29	0.92	0.93	1.38	3.33	1.08	2.08	1.07	2.25	2.82	4.63	2.93	0.61	1.72	2.57	3.25	3.57	1.68	0.87	0.68	1.05	1.80	—	—	0.59	4.23
9	Cinnamic acid	—	—	—	—	0.10	—	—	—	0.04	0.11	—	0.12	—	—	—	—	—	—	—	0.02	—	—	—	—	—	0.03
10	4-Methoxy cinnamic acid	0.36	—	0.30	—	—	—	—	—	—	0.72	0.62	0.63	0.51	—	0.36	0.21	0.30	0.49	0.28	0.25	0.25	0.31	—	—	—	0.23
11	5-Methoxy pinobanksin	—	—	0.40	0.78	1.88	0.44	—	—	1.21	3.04	1.73	2.14	0.56	—	2.59	0.31	0.50	0.64	0.60	0.81	—	0.62	—	—	—	0.43
12	Pinobanksin	13.11	8.19	6.01	7.98	9.21	2.98	10.53	8.12	10.24	20.60	15.37	17.63	3.19	4.49	6.34	12.89	10.06	14.13	10.15	2.13	2.00	7.64	4.43	9.54	0.75	5.67
13	Quercetin	—	0.26	0.42	0.50	0.47	—	7.57	0.58	0.34	0.43	0.51	—	—	—	0.25	0.30	0.19	—	0.26	—	—	0.42	0.04	—	—	0.25
14	Alpinetin	—	—	0.42	—	0.11	—	—	—	—	0.28	—	0.21	—	—	0.15	0.10	—	0.30	—	—	—	—	0.69	—	—	0.20
15	Kaempferol	0.86	0.51	0.18	0.51	0.50	0.28	0.61	0.43	0.47	0.93	0.63	0.75	0.21	0.21	0.46	0.44	0.42	0.62	0.39	0.23	0.21	0.41	0.29	—	—	0.18
16	Cinnamylideneacetic acid	0.68	0.24	0.16	0.44	1.03	—	0.32	0.40	0.62	—	0.31	0.16	—	—	0.42	—	0.37	—	—	—	—	0.31	—	—	—	—
17	Apigenin	2.45	—	—	—	2.12	0.65	—	—	2.09	—	1.68	—	0.56	0.56	1.90	0.58	0.78	1.72	0.99	0.48	0.65	1.64	—	—	—	0.45
18	Isorhamnetin	—	—	—	—	—	—	5.22	—	—	—	—	—	—	—	—	—	—	—	—	—	—	—	—	—	—	—
19	Pinocembrin	29.56	26.25	16.43	22.38	19.54	7.47	21.96	23.80	18.34	37.00	18.22	26.26	6.80	9.02	14.46	9.28	9.99	26.95	16.91	5.35	7.38	15.34	17.75	59.35	3.24	15.43
20	Benzyl caffeate	33.03	12.12	8.86	11.35	16.51	7.36	23.16	10.31	15.05	30.22	14.70	20.98	8.31	12.52	7.73	23.60	19.89	18.20	11.23	5.73	7.61	11.61	4.82	11.43	4.56	14.94
21	Pinobanksin-3-acetate	52.50	50.82	40.88	63.10	66.10	19.65	59.42	62.70	64.67	77.70	49.34	67.61	14.75	13.67	42.26	60.82	38.50	48.80	31.31	10.77	17.45	43.08	24.42	58.32	8.26	16.66
22	Chrysin	38.84	18.02	14.61	20.07	31.79	9.77	21.34	16.73	27.47	33.72	28.70	24.05	9.74	11.45	28.34	9.98	13.91	20.94	13.60	9.40	11.57	22.14	10.41	22.77	6.17	7.04
23	Phenethyl caffeate	27.53	5.64	5.48	9.22	11.00	3.05	10.74	6.89	7.29	12.18	10.97	18.76	1.67	15.35	7.66	10.96	6.43	11.67	6.73	1.80	3.55	4.42	4.23	2.90	0.91	23.11

24	Galangin	14.05	8.60	8.43	10.42	14.73	5.31	14.76	9.97	11.89	15.10	13.96	17.20	2.83	4.74	10.59	9.20	9.11	12.47	8.24	2.53	3.68	10.33	5.94	10.75	0.81	5.56
25	Benzyl *p*-coumarate	—	—	1.00	—	3.62	—	—	1.19	2.22	4.06	—	8.27	—	—	—	—	0.95	—	1.08	—	—	1.97	0.56	1.70	—	1.96
26	Benzyl ferulate	—	—	4.03	—	8.78	—	—	—	—	—	—	—	—	—	—	—	—	—	4.53	—	—	—	1.35	—	—	3.53
27	Cinnamyl caffeate	—	—	5.38	4.35	4.97	4.04	—	4.86	—	6.14	4.65	—	—	—	5.77	4.19	4.16	4.22	—	4.02	—	4.57	0.65	1.83	—	2.98
28	Pinostrobin	9.98	2.17	1.10	1.35	2.60	1.07	3.43	—	2.23	3.34	0.64	3.21	1.39	5.33	2.55	2.47	3.45	2.04	1.46	1.16	1.01	1.88	1.28	2.29	—	6.70
29	Tectochrysin	11.96	3.19	1.75	2.03	4.04	1.34	3.76	1.61	4.50	3.98	7.44	4.29	1.40	2.30	4.19	2.75	3.93	4.00	2.33	1.55	1.54	3.29	0.99	1.99	0.72	1.10
30	Cinnamyl *p*-cinnamate	—	—	—	3.51	4.06	2.71	—	6.56	5.42	—	—	—	—	2.95	—	3.13	—	—	—	—	—	5.67	0.51	2.00	—	—
31	Cinnamyl cinnamate	—	—	—	—	—	—	—	—	—	—	—	—	—	—	—	—	—	0.25	—	—	—	0.20	—	—	—	—
32	4-methoxy cinnamyl cinnamate	—	—	—	—	—	—	—	—	—	—	—	—	2.89	—	—	—	—	—	—	2.30	2.47	—	—	—	—	—
	SFC	173.31	118.01	90.63	129.12	153.09	48.96	148.6	123.94	143.45	196.12	138.22	163.35	41.43	51.77	114.08	109.12	90.84	132.61	86.24	34.41	45.49	106.79	66.24	165.01	19.95	59.67
	SPC	261.57	141.27	120.82	163.95	214.55	70.40	192.83	160.58	185.46	264.38	183.63	227.74	58.11	89.42	147.33	162.31	136.22	176.83	115.79	52.31	64.62	143.76	79.52	184.87	27.74	129.69

Note: "—": No detected. S18, S19 refer to Heilongjiang propolis; S20-S26 refer to Gansu propolis; S27 refers to Hebei propolis; S28, S29 refer to Liaoning propolis; S30 refers to Qinghai propolis; S31 refers to Shandong propolis; S32 refers to Shanxi propolis; S33, S34 refer to Shanxi propolis; S35, S36 refer to Sichuan propolis; S37 refers to Xizang propolis; S38, S39 refer to Xinjiang propolis and B2, B3, B4, B5 to refer to bud resins of *Populus ussuriensis* Kom., *Populus koreana* Rehd., *Populus cathayana* Rehd. and *Populus Simonii* × *P. nigra*, SFC represents the sum of flavonoid contents, SPC represents the sum of phenolic contents.

Table S3. Similarities of 17 propolis samples and bud resin of *Populus* × *euramericana* cv. ‘Neva’.

	Producing region	*Populus* × *euramericana cv.* ‘Neva’
S1	Henan	0.933
S2	Henan	0.923
S3	Henan	0.840
S4	Anhui	0.868
S5	Anhui	0.862
S6	Hebei	0.899
S7	Hubei	0.826
S8	Hubei	0.931
S9	Hubei	0.893
S10	Hubei	0.931
S11	Hubei	0.895
S12	Hunan	0.926
S13	Jilin	0.920
S14	Jiangsu	0.768
S15	Jiangsu	0.917
S16	Shandong	0.911
S17	Zhejiang	0.907

Table S4. Similarity evaluation of 17 propolis samples.

	S1	S2	S3	S4	S5	S6	S7	S8	S9	S10	S11	S12	S13	S14	S15	S16	S17	R
S1	1	0.949	0.886	0.942	0.933	0.860	0.939	0.929	0.906	0.950	0.940	0.878	0.935	0.946	0.897	0.965	0.973	0.967
S2	0.949	1	0.928	0.956	0.963	0.927	0.946	0.939	0.938	0.932	0.939	0.912	0.877	0.960	0.936	0.939	0.944	0.973
S3	0.886	0.928	1	0.931	0.952	0.956	0.918	0.916	0.869	0.913	0.922	0.949	0.844	0.942	0.941	0.907	0.893	0.954
S4	0.942	0.956	0.931	1	0.957	0.892	0.973	0.970	0.943	0.968	0.960	0.922	0.898	0.963	0.926	0.972	0.940	0.984
S5	0.933	0.963	0.952	0.957	1	0.901	0.951	0.938	0.943	0.931	0.949	0.944	0.848	0.963	0.946	0.934	0.951	0.980
S6	0.860	0.927	0.956	0.892	0.901	1	0.868	0.864	0.822	0.860	0.864	0.874	0.834	0.906	0.899	0.860	0.856	0.909
S7	0.939	0.946	0.918	0.973	0.951	0.868	1	0.965	0.946	0.947	0.939	0.950	0.860	0.94	0.945	0.959	0.925	0.978
S8	0.929	0.939	0.916	0.970	0.938	0.864	0.965	1	0.907	0.983	0.940	0.909	0.895	0.945	0.905	0.966	0.928	0.972
S9	0.906	0.938	0.869	0.943	0.943	0.822	0.946	0.907	1	0.895	0.923	0.905	0.799	0.934	0.907	0.913	0.901	0.947
S10	0.950	0.932	0.913	0.968	0.931	0.860	0.947	0.983	0.895	1	0.958	0.887	0.927	0.956	0.886	0.983	0.948	0.973
S11	0.940	0.939	0.922	0.960	0.949	0.864	0.939	0.940	0.923	0.958	1	0.908	0.895	0.971	0.910	0.968	0.951	0.976
S12	0.878	0.912	0.949	0.922	0.944	0.874	0.950	0.909	0.905	0.887	0.908	1	0.782	0.915	0.962	0.892	0.873	0.947
S13	0.935	0.877	0.844	0.898	0.848	0.834	0.860	0.895	0.799	0.927	0.895	0.782	1	0.905	0.810	0.936	0.928	0.911
S14	0.946	0.960	0.942	0.963	0.963	0.906	0.940	0.945	0.934	0.956	0.971	0.915	0.905	1	0.937	0.957	0.948	0.983
S15	0.897	0.936	0.941	0.926	0.946	0.899	0.945	0.905	0.907	0.886	0.910	0.962	0.810	0.937	1	0.895	0.889	0.954
S16	0.965	0.939	0.907	0.972	0.934	0.860	0.959	0.966	0.913	0.983	0.968	0.892	0.936	0.957	0.895	1	0.962	0.979
S17	0.973	0.944	0.893	0.940	0.951	0.856	0.925	0.928	0.901	0.948	0.951	0.873	0.928	0.948	0.889	0.962	1	0.968
R	0.967	0.973	0.954	0.984	0.98	0.909	0.978	0.972	0.947	0.973	0.976	0.947	0.911	0.983	0.954	0.979	0.968	1

Table S5. Similarities of 22 propolis samples and bud resins of four poplar.

	Producing region	*Populus ussuriensis* Kom.	*Populus koreana* Rehd.	*Populus cathayana* Rehd.	*Populus Simonii* × *P. nigra*
S18	Heilongjiang	0.829	0.777	0.801	0.966
S19	Heilongjiang	0.951	0.920	0.844	0.934
S20	Gansu	0.935	0.885	0.848	0.971
S21	Gansu	0.923	0.866	0.847	0.963
S22	Gansu	0.881	0.802	0.872	0.973
S23	Gansu	0.905	0.840	0.864	0.940
S24	Gansu	0.893	0.835	0.824	0.975
S25	Gansu	0.931	0.882	0.825	0.960
S26	Gansu	0.891	0.811	0.877	0.969
S27	Hebei	0.930	0.885	0.868	0.863
S28	Liaoning	0.864	0.791	0.843	0.919
S29	Liaoning	0.880	0.824	0.809	0.874
S30	Qinghai	0.858	0.802	0.895	0.765
S31	Shandong	0.741	0.691	0.695	0.916
S32	Shanxi	0.857	0.779	0.887	0.875
S33	Shanxi	0.715	0.639	0.656	0.872
S34	Shanxi	0.788	0.715	0.749	0.945
S35	Sichuan	0.933	0.896	0.821	0.952
S36	Sichuan	0.936	0.897	0.824	0.866
S37	Xizang	0.853	0.790	0.914	0.950
S38	Xinjiang	0.877	0.815	0.875	0.970
S39	Xinjiang	0.896	0.826	0.873	0.606

Table S6. Similarity evaluation of 22 propolis samples.

	S18	S19	S20	S21	S22	S23	S24	S25	S26	S27	S28	S29	S30	S31	S32	S33	S34	S35	S36	S37	S38	S39	R
S18	1	0.973	0.963	0.973	0.940	0.975	0.959	0.969	0.869	0.967	0.934	0.922	0.874	0.770	0.916	0.842	0.872	0.948	0.952	0.866	0.950	0.975	0.981
S18	0.973	1	0.951	0.954	0.972	0.983	0.970	0.955	0.843	0.969	0.903	0.942	0.868	0.740	0.930	0.815	0.911	0.961	0.963	0.854	0.911	0.939	0.978
S19	0.963	0.951	1	0.986	0.934	0.936	0.973	0.965	0.902	0.932	0.965	0.912	0.875	0.805	0.950	0.823	0.885	0.921	0.924	0.886	0.960	0.979	0.981
S20	0.973	0.954	0.986	1	0.942	0.943	0.963	0.972	0.916	0.947	0.951	0.922	0.877	0.824	0.951	0.816	0.890	0.942	0.944	0.886	0.968	0.974	0.986
S21	0.940	0.972	0.934	0.942	1	0.945	0.945	0.938	0.859	0.953	0.891	0.966	0.845	0.769	0.935	0.782	0.938	0.967	0.966	0.839	0.890	0.918	0.971
S22	0.975	0.983	0.936	0.943	0.945	1	0.967	0.951	0.834	0.973	0.890	0.926	0.858	0.724	0.889	0.821	0.881	0.942	0.947	0.836	0.894	0.936	0.967
S23	0.959	0.970	0.973	0.963	0.945	0.967	1	0.956	0.871	0.946	0.938	0.925	0.882	0.758	0.951	0.808	0.903	0.932	0.937	0.886	0.934	0.964	0.980
S24	0.969	0.955	0.965	0.972	0.938	0.951	0.956	1	0.891	0.962	0.942	0.933	0.874	0.803	0.923	0.798	0.869	0.952	0.956	0.873	0.936	0.960	0.981
S25	0.869	0.843	0.902	0.916	0.859	0.834	0.871	0.891	1	0.88	0.885	0.867	0.814	0.878	0.885	0.700	0.832	0.876	0.876	0.843	0.914	0.891	0.917
S26	0.967	0.969	0.932	0.947	0.953	0.973	0.946	0.962	0.880	1	0.891	0.954	0.870	0.773	0.897	0.786	0.858	0.954	0.956	0.860	0.902	0.928	0.971
S27	0.934	0.903	0.965	0.951	0.891	0.89	0.938	0.942	0.885	0.891	1	0.882	0.861	0.797	0.920	0.788	0.851	0.898	0.904	0.880	0.944	0.969	0.953
S28	0.922	0.942	0.912	0.922	0.966	0.926	0.925	0.933	0.867	0.954	0.882	1	0.828	0.793	0.899	0.769	0.889	0.935	0.937	0.819	0.869	0.900	0.956

S29	0.874	0.868	0.875	0.877	0.845	0.858	0.882	0.874	0.814	0.870	0.861	0.828	1	0.713	0.874	0.684	0.777	0.859	0.864	0.978	0.876	0.882	0.892
S30	0.770	0.740	0.805	0.824	0.769	0.724	0.758	0.803	0.878	0.773	0.797	0.793	0.713	1	0.780	0.617	0.714	0.779	0.776	0.741	0.821	0.790	0.818
S31	0.916	0.930	0.950	0.951	0.935	0.889	0.951	0.923	0.885	0.897	0.92	0.899	0.874	0.780	1	0.722	0.915	0.939	0.936	0.903	0.946	0.938	0.957
S32	0.842	0.815	0.823	0.816	0.782	0.821	0.808	0.798	0.700	0.786	0.788	0.769	0.684	0.617	0.722	1	0.765	0.746	0.755	0.657	0.787	0.819	0.827
S33	0.872	0.911	0.885	0.89	0.938	0.881	0.903	0.869	0.832	0.858	0.851	0.889	0.777	0.714	0.915	0.765	1	0.93	0.929	0.774	0.844	0.863	0.920
S34	0.948	0.961	0.921	0.942	0.967	0.942	0.932	0.952	0.876	0.954	0.898	0.935	0.859	0.779	0.939	0.746	0.930	1	0.998	0.856	0.906	0.920	0.969
S35	0.952	0.963	0.924	0.944	0.966	0.947	0.937	0.956	0.876	0.956	0.904	0.937	0.864	0.776	0.936	0.755	0.929	0.998	1	0.861	0.908	0.927	0.972
S36	0.866	0.854	0.886	0.886	0.839	0.836	0.886	0.873	0.843	0.860	0.880	0.819	0.978	0.741	0.903	0.657	0.774	0.856	0.861	1	0.903	0.893	0.894
S37	0.950	0.911	0.960	0.968	0.890	0.894	0.934	0.936	0.914	0.902	0.944	0.869	0.876	0.821	0.946	0.787	0.844	0.906	0.908	0.903	1	0.970	0.957
S38	0.975	0.939	0.979	0.974	0.918	0.936	0.964	0.960	0.891	0.928	0.969	0.900	0.882	0.790	0.938	0.819	0.863	0.92	0.927	0.893	0.970	1	0.975
S39	0.981	0.978	0.981	0.986	0.971	0.967	0.980	0.981	0.917	0.971	0.953	0.956	0.892	0.818	0.957	0.827	0.92	0.969	0.972	0.894	0.957	0.975	1
